# Enhancing Low Back Pain Management: The Diagnostic and Prognostic Role of Single Photon Emission Computed Tomography-Computerized Tomography (SPECT-CT) in Surgical Outcomes

**DOI:** 10.7759/cureus.84015

**Published:** 2025-05-13

**Authors:** Brittany G Futch, Vishal Venkatraman, Ellen OCallaghan, Jessica Albanese, Samah M Morsi, Mounica Paturu, Marie Futrell, Norah Foster, Brett Rocos, Mazen Zein, Khoi D Than, Christopher Shaffrey, C. Rory Goodwin, Colm Kelleher, Muhammad Abd-El-Barr

**Affiliations:** 1 Neurosurgery, Duke University Medical Center, Durham, USA; 2 Orthopedic Surgery, Duke University Medical Center, Durham, USA; 3 Orthopedic Surgery, Premier Health, Centerville, USA; 4 Radiology, Duke University Medical Center, Durham, USA

**Keywords:** chronic low back pain (clbp), lumbar interbody fusion, lumbar spine pain, spect-ct, spect-ct bone scintigraphy spinal hot spots nuclear imaging spinal imaging spinal diagnostics

## Abstract

Purpose: How specific active areas on Single Photon Emission Computed Tomography-Computerized Tomography (SPECT-CT) inform management for chronic low back pain (CLBP) is limited. This study aimed to assess the relationship between SPECT-CT uptake locations with spinopelvic parameters, Pfirmann grades, and Modic changes, and to evaluate whether these locations correlate with pain outcomes after surgery.

Methods: A retrospective analysis of 26 patients who underwent SPECT-CT followed by interbody fusion from January 2018 to January 2023 was conducted. Patients were categorized based on uptake patterns in the lumbar spine: disc space only, facet joint only, or both. Key outcomes included Visual Analog Scale (VAS) pain scores at one, three, six, and 12-month intervals. Statistical analyses were performed to assess correlations and pain score changes over time.

Results: Of 26 patients, 38.5% exhibited uptake in the disc space, 23.0% in the facet joint, and 38.5% in both. Significant pain reductions were noted in those with disc space uptake (p = 0.016), achieving minimal clinically important difference (MCID) thresholds by six months. In contrast, facet joint uptake showed no clinically significant pain relief until 12 months, while simultaneous uptake in both areas resulted in statistically significant improvements at one, three, and 12 months (p = 0.005, 0.001, 0.032, respectively).

Conclusion: SPECT-CT uptake in the disc space is associated with improved postoperative outcomes in CLBP, while isolated facet joint uptake does not yield significant pain relief. This study highlights the diagnostic and prognostic value of SPECT-CT in guiding surgical decision-making and improving patient outcomes.

## Introduction

While traditional imaging techniques, such as magnetic resonance imaging (MRI) and computed tomography (CT), are considered the standard of care for localizing possible pain generators in chronic low back pain, the etiology remains unknown for a substantial portion of affected individuals. This has led to the classification of many cases as “nonspecific low back pain” [1]. Therefore, novel diagnostic modalities capable of identifying potential pain generators are essential.

Single Photon Emission Computed Tomography-Computerized Tomography (SPECT-CT) has demonstrated effectiveness in diagnosing pain generators when conventional imaging methods yield inconclusive results [2]. This is primarily due to SPECT-CT’s ability to provide both anatomical and functional information. For example, in 1992, Ryan et al. demonstrated that of 54 lesions detected by SPECT, only 37% were seen on planar imaging [3]. Additionally, SPECT-CT has been shown to detect anatomical changes earlier than MRI, including early endplate changes, and can differentiate between incidental and clinically significant MRI findings [4,5]. When SPECT-CT is employed for diagnosing potential pain generators, patients have reported improvements in pain outcomes [6]. Furthermore, SPECT-CT can aid treating physicians in directing patients to appropriate treatment modalities, including intra-articular facet injections [6,7] and surgical interventions [8,9]. In 2017, Russo et al. demonstrated that SPECT-CT may detect early facet joint deterioration [10], and in 2019, Tender et al. reported significant improvement in pain for patients receiving surgery based on SPECT-CT findings [11]. In addition to clinical factors, economic and practical considerations must also be taken into account. For example, according to GoodRx, the high-end cost of an MRI is approximately $12,000, whereas the high-end cost of a SPECT-CT is around $6,750 [12,13]. SPECT-CT is also far less time-consuming than MRI and is not prohibitive if patients have certain implants, such as pacemakers or prior instrumentation [14].

Objective

While existing literature on SPECT-CT supports its diagnostic utility, research on how specific uptake areas can inform the management of low back pain remains limited. Furthermore, it is unclear whether SPECT-CT findings provide additional insights beyond those available from traditional imaging, such as radiographs and MRI. This study aims to investigate: 1) the association between specific uptake sites and spinopelvic parameters on radiographs, as well as Pfirmann grades and Modic changes on MRI; and 2) whether these uptake sites correlate with an increased likelihood of improved pain outcomes following surgery.

## Materials and methods

Sample selection

A retrospective analysis conducted at a single academic institution identified 35 patients who underwent SPECT-CT (isotope technetium-99m (99mTc)) for low back pain and/or lumbar radiculopathy, followed by interbody fusions from January 2018 to January 2023. To minimize confounding from spondylolisthesis, we also excluded patients who had radiotracer uptake and subsequent fusion at a level with pre-existing spondylolisthesis, resulting in a final sample size of 26 patients.

Data extraction

Data collected included patient age, gender, uptake patterns, spinopelvic parameters, Pfirmann grades, Modic changes, and baseline pain scores on the visual analogue scale. Spinopelvic parameters included sacral vertical axis (SVA), lumbar lordosis (LL), pelvic incidence (PI), pelvic tilt (PT), and sacral slope (SS) as defined in a 2018 review [15]. The sacral vertical axis was performed on lateral standing full-length spine X-rays. A line is drawn vertically from the middle of the C7 vertebral body to S1, and the distance is measured. Pelvic incidence was the angle created by intersecting the midpoint of the femoral heads to the midpoint of the superior endplate of the sacrum and a line perpendicular to the superior endplate of the sacrum. Pelvic tilt is the distance between the midportion of the sacrococcygeal joint and the upper border of the symphysis pubis. The sacral slope was defined as the angle between a line drawn parallel to the superior endplate of S1 and a horizontal reference.

Pfirmann Grades and Modic changes were defined based on their original conceptions as defined in the literature in the 1970s [16] and 1980s [17], respectively. Pfirmann grades relate specifically to changes of the intervertebral disc, whereas Modic changes are specific to superior and inferior end plate changes surrounding the disc space. Pfirmann grades were on a scale ranging from I-V, with V demonstrating the highest degree of pathology (completely collapsed). Modic changes included type 1-3 changes, with type 1 representing inflammatory changes, type 2 as lipid replacement of the marrow, and type 3 demonstrating calcification of the endplates. Outcomes assessed for these patients included Visual Analogue Scale (VAS) pain scores at one, three, six, and 12 months following surgery.

Statistical analysis

Patient demographics, including age, were summarized using means and standard deviations, while gender was reported as percentages. Mean spinopelvic parameters were provided for each uptake location, along with counts for each Pfirmann grade and type of Modic change. VAS scores for pain improvement were summarized at each time point using means and standard deviations. Comparisons with ANOVA were used for spinopelvic parameters across the three different uptake signatures. Similarly, we compared Pfirmann grades and Modic changes using the Chi-square test. Comparisons of VAS pain scores within groups over time were performed using PyQt5 version 5.9.2 and version 5.9.7 ( https://www.anaconda.com).

## Results

Baseline characteristics

**Figure 1:**
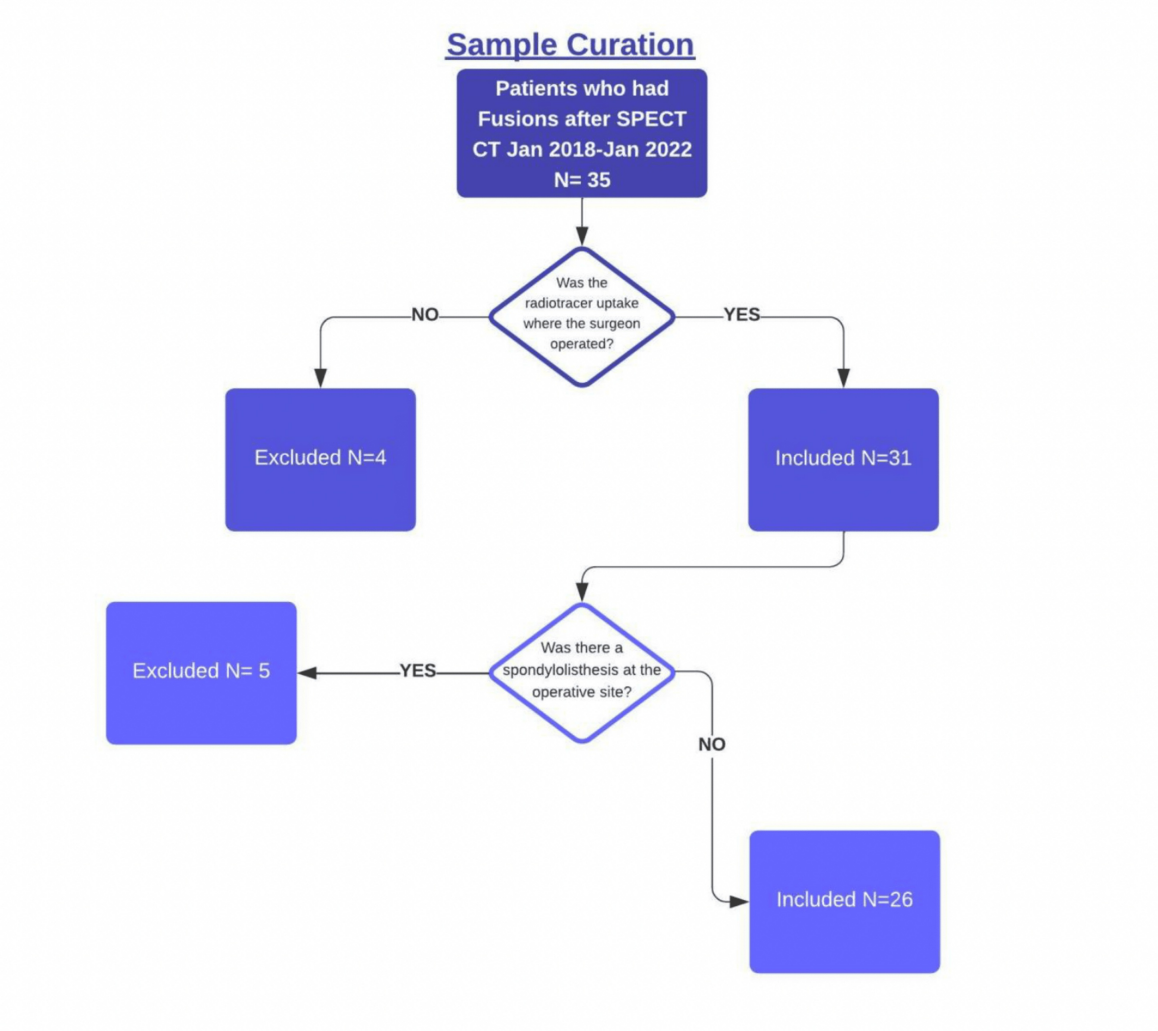
Flow chart of patient selection SPECT-CT: Single Photon Emission Computed Tomography-Computerized Tomography

From January 2018 to January 2023, we identified 26 patients who had interbody fusions performed at a level that demonstrated radiotracer uptake without spondylolisthesis (Figure 1).

**Figure 2:**
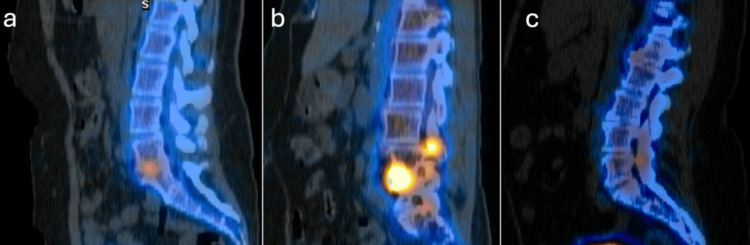
Examples of different SPECT-CT signatures a) Disc space only: 48-year-old female. Single Photon Emission Computed Tomography-Computerized Tomography (SPECT-CT) dominant uptake at the L5/S1 disc space and had a baseline visual analogue scale (VAS) pain score of 7. After an L5/S1 anterior lumbar interbody fusion (ALIF), she reported a VAS pain score of 2 at one year postoperatively. b) Disc space and facet joint: 69-year-old. SPECT-CT dominant uptake at L4/L5 disc space and L3/4 facet joint with a baseline VAS pain score of 6. She underwent an L3-L5 lateral interbody fusion and reported a VAS pain score of 4 at one year postoperatively. c) Facet joint only: 58-year-old female. SPECT-CT dominant uptake at the L4/L5 facet joint and had a baseline VAS pain score of 7. She underwent an L4/L5 prone trans-psoas approach interbody fusion along with L4/L5 posterior instrumentation and reported a VAS pain score of 6 at one year postoperatively.

Looking carefully at the uptake patterns, we found three main areas of uptake: uptake in the disc space only, facet joint only, or both a facet joint and a disc space simultaneously. Figure 2 demonstrates an example of these three different signatures. It is important to clarify that "uptake within the disc space" refers specifically to uptake in the end plates, not the disc itself, in accordance with standard radiology terminology.

We found that 10/26 (38.5%) of patients had uptake within the disc space, 6/26 (23.0%) had uptake in the facet joint, and 10/26 (38.5%) had uptake in both the disc space and facet joint (Table 1). The mean age of the disc space only group was 60.1 +/- 16.16 years, the facet group was 66.17 +/- 7.03 years, and the both group was 60.5 +/- 8.3 years. An ANOVA comparing mean age across the three uptake signatures found no statistically significant differences (p=0.57). We found similar nonsignificant results comparing BMI and sex across the three uptake signatures using an ANOVA and Chi-square statistic, respectively (p=0.51 and p=0.79).

**Table 1:** Baseline demographics

Uptake Location	Age (mean)	Age (std)	BMI (mean)	BMI (std)	Female (%)	Total	Age p-value	BMI p-value	Sex p-value
Disc Space Only	60.1	16.16	30.27	5.24	50.0	10	0.57	0.51	0.79
Facet Joint Only	66.17	7.03	27.31	4.03	66.67	6			
Both	60.5	8.3	28.43	5.4	60.0	10			

Spinopelvic parameters, Pfirmann grades, and Modic changes

**Figure 3:**
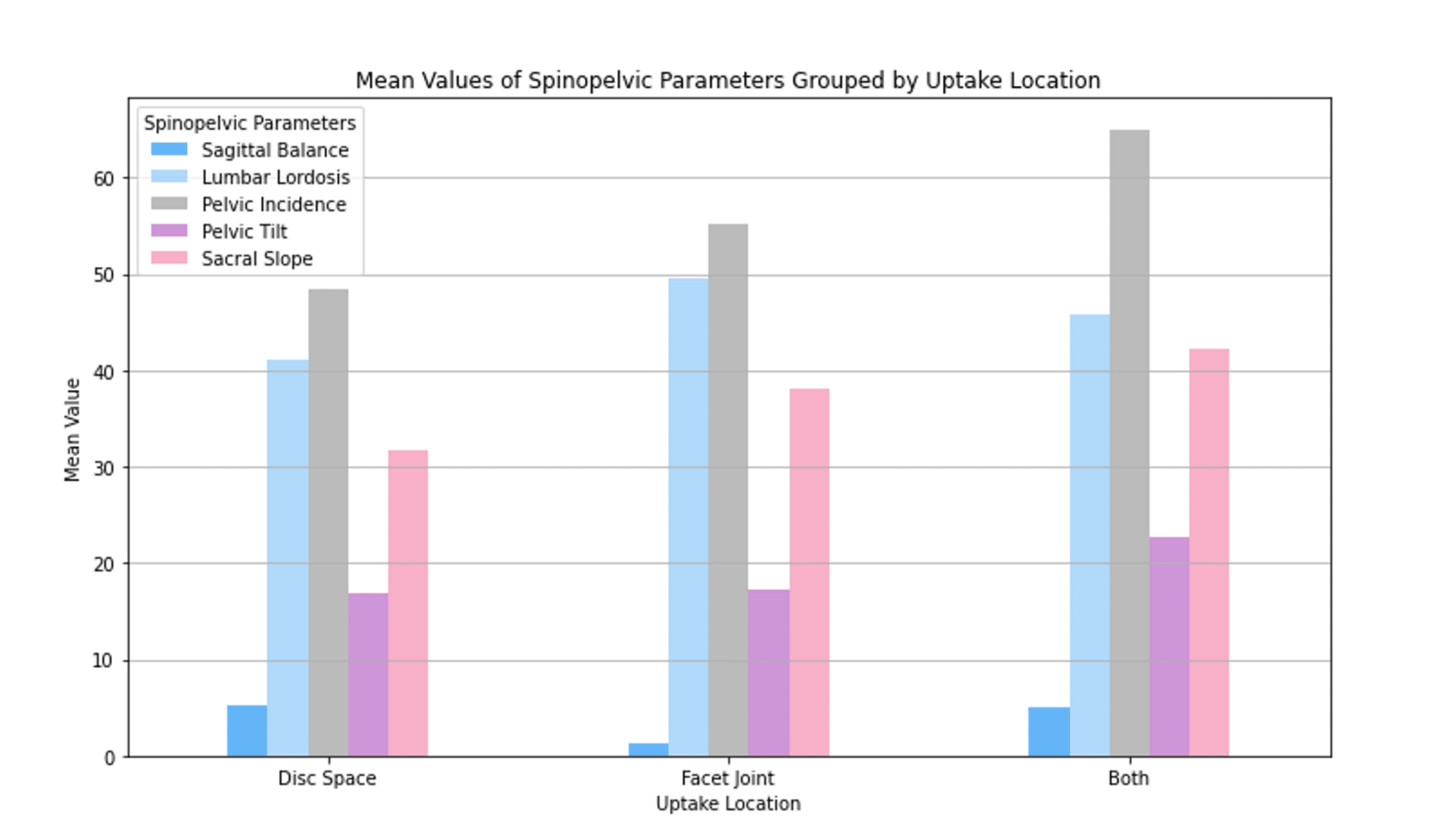
Differences in spinopelvic parameters based on SPECT-CT uptake location SPECT-CT: Single Photon Emission Computed Tomography-Computerized Tomography

**Figure 4:**
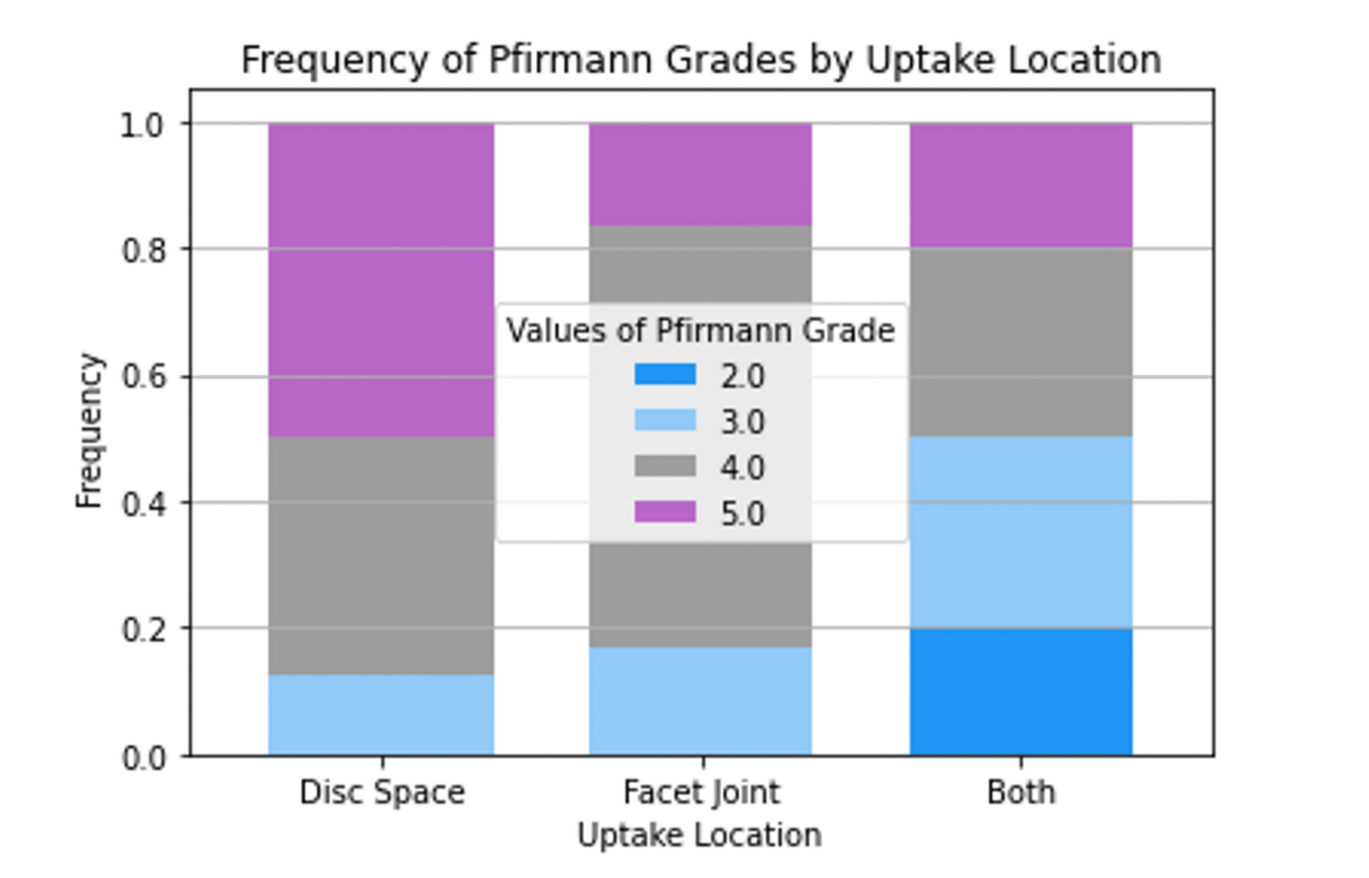
Differences in Pfirmann grades based on SPECT-CT uptake location SPECT-CT: Single Photon Emission Computed Tomography-Computerized Tomography

**Figure 5:**
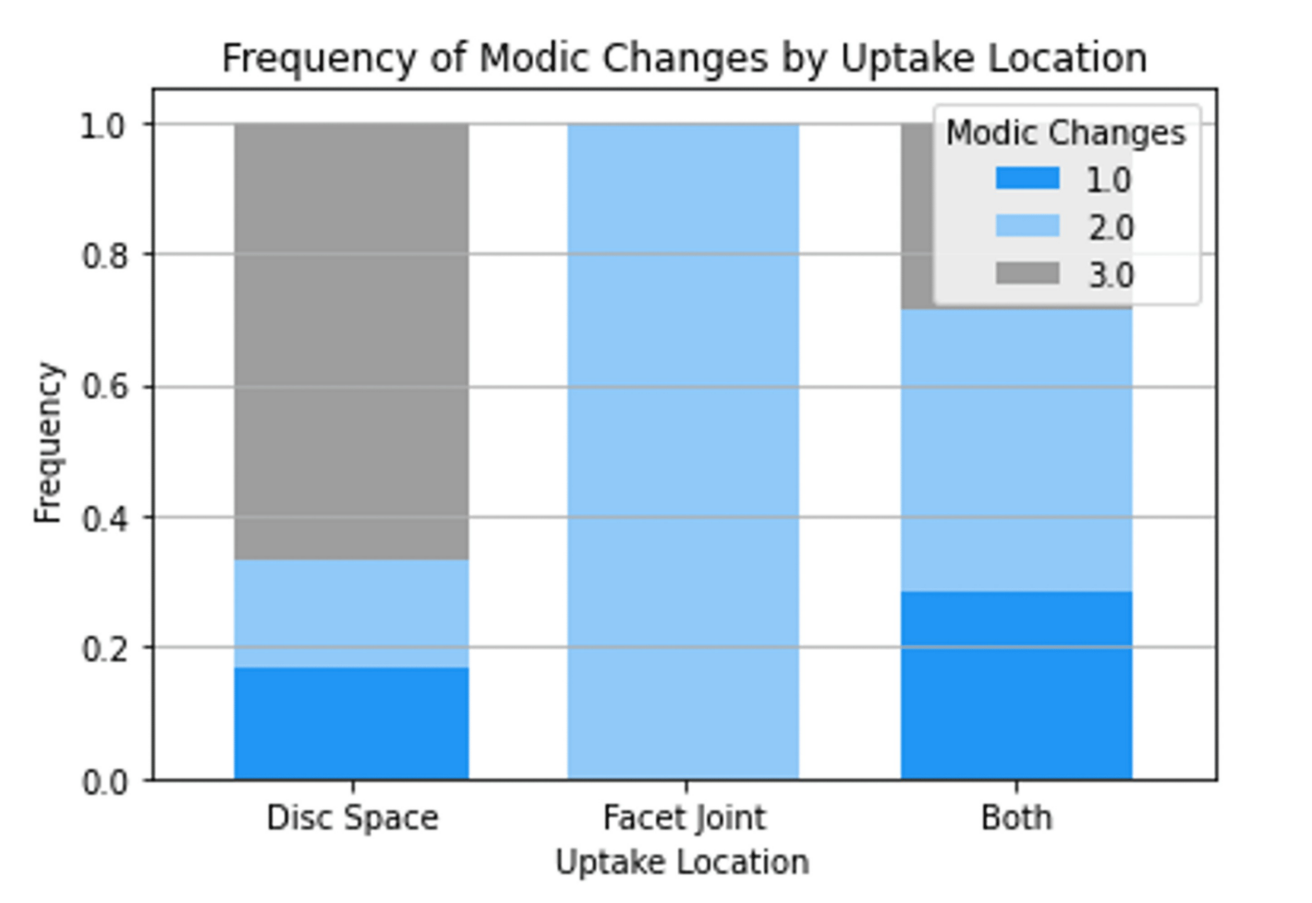
Differences in Modic Changes based on SPECT-CT uptake location SPECT-CT: Single Photon Emission Computed Tomography-Computerized Tomography

Mean spinopelvic parameters, including sagittal balance, lumbar lordosis (LL), pelvic incidence (PI), pelvic tilt (PT), and sacral slope (SS), were calculated and presented according to uptake location (Figure 3). The mean values for each spinopelvic parameter were comparable across all three uptake locations and did not demonstrate statistical significance. Raw counts were collected for Pfirmann Grades one to five at each uptake site (Figure 4). Notably, no patients exhibited a Pfirmann grade of one. Only patients with uptake in both the disc space and facet joint simultaneously showed a Pfirmann grade of two. Pfirmann grades three to five were compared across all three uptake locations and were not statistically different (p=0.361). Additionally, raw counts for Modic changes were documented for each uptake site (Figure 5). Patients with uptake restricted to the facet joint did not display type one Modic changes. Those with uptake solely in the disc space had the lowest counts of type two Modic changes, while type two Modic changes were comparable in patients with only facet joint uptake and those with simultaneous uptake in both locations. Patients with facet joint uptake did not exhibit type three Modic changes, whereas disc space uptake showed the highest frequency of type three Modic changes. Again, statistical analysis comparing counts of Modic changes across uptake sites was not determined to be statistically significant (p=0.150).

Uptake location and pain scores over time

**Figure 6:**
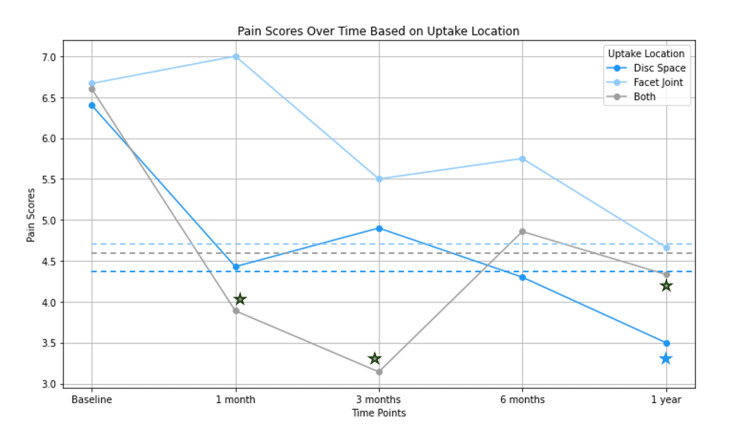
Pain scores over time based on SPECT-CT uptake location SPECT-CT: Single Photon Emission Computed Tomography-Computerized Tomography

Patients exhibiting predominant uptake in the disc space showed significant reductions in overall mean VAS pain scores following posterior lumbar interbody fusions, with results observed at 12 months postoperatively (p = 0.016) (Figure 6). According to existing literature, a minimal clinically important difference (MCID) of 2.0 units in VAS pain scores is required for patients undergoing spinal fusion to be considered clinically improved [18]. Those with disc space uptake achieved the MCID at both six and 12 months after surgery. Conversely, patients with radiotracer activity confined to the facet joint did not exhibit significant improvements in their pain scores postoperatively; however, they did reach the MCID threshold at the 12-month follow-up. In patients demonstrating simultaneous radiotracer activity in both the disc space and facet joint, there was a statistically significant improvement in mean VAS pain scores at one, three, and 12 months post-surgery (p = 0.005, p = 0.001, and p = 0.032, respectively). These improvements also met the MCID criteria.

## Discussion

Although the lifetime prevalence of chronic low back pain remains high within the United States, diagnosis of potential etiologies remains a challenge [19]. This is largely due to the variety of sources of low back pain (i.e., axial lumbosacral, radicular, and referred pain) [20]. To further complicate the diagnostic process, axial lumbosacral pain can be further categorized based on anatomic pain generation site, including myofascial pain, facet joint pain, sacroiliac joint pain, discogenic pain, spinal stenosis, and failed back surgery [19].

Diagnosing and differentiating between facet joint pain and discogenic pain is extremely difficult. This is partially because as the spine degenerates, these anatomical structures will necessarily degenerate, yet not necessarily be pain generators. Moreover, it is unclear whether the treatments of pain from these different sources should be different. Current methods to diagnose pain generator site involve an in-depth patient history, including onset and duration of the pain as well as characterization (i.e., burning, aching, numbness, and electric shock sensations) and provoking factors (sitting, standing, walking, and lying down). Various physical exam maneuvers, including the Patrick’s test [21], a straight leg test [22], and a Gaenslen’s test [23], can also be employed to further distinguish possible anatomical locations generating pain. Unfortunately, in most cases, history and physical alone are insufficient, often requiring further workup with electrodiagnostics and imaging.

Imaging modalities employed to diagnose low back pain generators range from simple radiographs depicting planar bony anatomy as well as detailed 3D imagery of soft tissues on MRI. Most physicians begin with X-rays given its ability to asses alignment as well as instability with dynamic imaging performed with the patient in flexion and extension [24]. CT or MRI is later pursued if there is suspicion for complex bony pathology or nerve compression, respectively [24,25].

SPECT-CT provides a potential avenue for improved and more precise localization of pain generation through its ability to provide both detailed anatomical bony information as seen on CT as well as metabolic activity as seen on PET [26]. Unfortunately, most studies examining the utility of SPECT-CT for diagnosing pain generators in the lumbar spine often lack granularity, as they frequently do not specify the location of radiotracer activity within spinal structures.

Our study is the first known one to demonstrate that SPECT-CT patients can have distinct areas of uptake within the lumbar spine while having comparable findings on both radiographs and MRI (i.e., spinopelvic parameters, Pfirmann grades, and Modic changes). Additionally, depending on the dominant site of radiotracer activity, patients may have different long-term pain outcomes after receiving interbody fusions. Specifically, for patients who have dominant uptake within the disc space, these patients have statistically significant improvements in their pain scores over the course of a year after minimally invasive lumbar interbody fusions. Patients with both disc space and facet joint uptake similarly have improvements in their pain scores, though to a lesser extent. Unfortunately for patients who have uptake within the facet joint, we did not see a statistically significant improvement in their pain after receiving interbody fusions. We will discuss each of these results in more depth and compare these results with the existing (albeit limited) literature in the following sections.

Baseline characteristics

A wide variety of uptake signatures appears to be reported in the literature. It is unclear whether the distribution of uptake locations (disc space versus facet joint) represents the difference between discogenic vs facetogenic back pain. This is largely partly due to the lack of granularity of existing literature as well as the current inability to accurately and consistently attribute back pain as being discogenic or facetogenic in nature [27,28]. To further compound this issue, prior research also tends to present findings in isolation without correlating them with MRI results at the corresponding levels or considering the presence of spondylolisthesis. Therefore, we are unable to say whether the larger population does in fact have majority discogenic-related pain with a degree of appreciable Pfirmann grades, Modic changes, and/or SPECT-CT scintigraphy. Larger studies are needed to accurately assess the prevalence of uptake in the disc space versus the facet joint.

SPECT-CT provides additional information not detected on radiographs or MRI

Before analyzing pain scores over time based on uptake location, this study aimed to describe differences in spinopelvic parameters, Pfirmann grades, and Modic changes associated with each uptake location. We performed these assessments because research has consistently demonstrated that Sagittal spino-pelvic alignment, Pfirmann grades, and Modic changes between patients with chronic low back pain and healthy controls are statistically different [29,30]. We also excluded patients from our study who had a pre-existing spondylolisthesis, given prior research demonstrating modest clinical improvements in patients who undergo surgery at that level compared to those who did not undergo surgery [31].

By assessing anatomical variations identified through X-rays and MRIs based on SPECT-CT uptake, we could ascertain whether SPECT-CT offers additional diagnostic information not captured by traditional imaging modalities. In other words, if patients demonstrate similar spinopelvic parameters, Pfirmann grades, and Modic changes, yet demonstrate distinct radiotracer signatures on SPECT-CT, we have increased confidence that SPECT-CT provides the diagnostician with novel information. Results from our study did in fact indicate that spinopelvic parameters, Pfirmann grades, and Modic changes were not statistically significantly different between specific uptake locations on SPECT-CT. Consequently, we may be able to assume that supplementary information obtained from SPECT-CT does not overlap with findings visible on conventional imaging.

Our provided case examples (Figure 2) as well as our overall results align with previous research suggesting that SPECT-CT may more accurately identify pain generators compared to traditional imaging [32]. A recent 2023 study demonstrated that patients undergoing interventions based on SPECT-CT localized pain generators experienced a 70.59% reduction in pain scores, while those diagnosed via MRI reported only a 58.57% improvement following intervention [32]. Moreover, a 2024 study indicated that X-rays and MRI identified pain generators in 13.0% and 29.7% of cases, respectively, whereas SPECT-CT identified them in 57.8% of cases [33].

Disc spaces with active scintigraphy improve after interbody fusion

Following the confirmation that SPECT-CT provided unique information compared to radiographs and MRI, we examined how pain scores evolved over time based on uptake location with interbody fusion. Notable improvements in mean VAS pain scores were observed in patients with uptake patterns predominantly located in the disc space or simultaneously in both the disc space and facet joint (see Figure 2 for case examples). This finding is particularly pertinent given the challenges associated with diagnosing and treating discogenic back pain. For instance, discography, also referred to as discograms, has been utilized to evaluate refractory low back pain; however, this technique remains controversial due to variability in methodology and diagnostic criteria.

In contrast, those patients who had increased uptake in the facet joint alone showed less relief following interbody fusions compared to those who had uptake in the disc space alone or disc space and facet joint after minimally invasive lumbar interbody fusions. This may be because in minimally invasive (MIS) interbody fusions, most of the stabilization happens in the disc space, rather than in the inter-transverse ligament (see Figure 2 for case example) [34,35]. Moreover, there has been increasing evidence that facet fusion may be additive in typical minimally invasive transforaminal lumbar interbody fusion (MIS-TLIF) [36].

Limitations

A significant limitation of this study is that SPECT-CT is primarily regarded as a qualitative imaging modality rather than a quantitative one [37,38]. Currently, there is no standardized grading scale in clinical practice that reliably categorizes uptake on SPECT-CT as mild, moderate, or severe. Consequently, there is a risk that imaging for patients with simultaneous uptake in both the disc space and facet joint may be interpreted differently by various radiologists, with one potentially classifying it as primarily disc space uptake and another as facet joint uptake. Future research should focus on developing a method to quantify SPECT-CT activity in a manner analogous to Hounsfield units (HU) in CT scans or standardized uptake values (SUV) in PET scans. This study was additionally limited in that only one type of intervention was investigated, namely the use of MIS lumbar interbody fusions. It would be interesting to see how the patients with different SPECT-CT signatures fared after different interventions, such as targeted injections.

Additionally, while the sample size in this study is comparable to that of other investigations, its statistical power remains limited. Furthermore, this study does not function as a predictive model; thus, factors such as smoking status and other baseline comorbidities were not controlled for. To ascertain the predictive utility of SPECT-CT in identifying pain generators, it would be beneficial to conduct controlled prospective studies or larger studies incorporating machine learning techniques.

## Conclusions

The present study findings indicate that there are different signatures for SPECT-CT uptake in patients with low back pain. These signatures may represent different etiologies of the pain generator, namely discogenic and facet-mediated pain. Interestingly, patients exhibiting uptake in the disc space experienced a reduction in low back pain following interbody fusion procedures, while patients with uptake limited to the facet joint did not show this improvement. Larger, randomized control studies are needed to better understand the use of SPECT-CT to diagnose and direct treatment for patients with low back pain.
